# Surgical management of an adult patient with Cataract and bilateral Ectopia Lentis: a case report

**DOI:** 10.22336/rjo.2021.54

**Published:** 2021

**Authors:** Maria-Monica Gavriș, Alexandru Kun, Iulia-Maria Gavriș

**Affiliations:** * “Constantin Papilian” Military Emergency Hospital Cluj-Napoca, Romania; ** “Iuliu Hațieganu” University of Medicine and Pharmacy Cluj-Napoca, Romania

**Keywords:** Ectopia Lentis, the new capsule retractors, CTR with scleral suture

## Abstract

A 48-year-old female patient presented to the Ophthalmology Clinic with gradual loss of vision in both eyes over the last year. Upon slit lamp examination, cortical and nuclear opacities of the lens in BE and a 180-degree zonular dehiscence with moderate bilateral superotemporal dislocation of the lens were revealed. After a series of necessary investigations, the patient was admitted for surgery. This case report represents an addition to the diagnosis, challenges and the latest surgical techniques and instruments.

## Introduction

Ectopia Lentis defines the ocular pathology, hereditary or acquired in most cases, which involves the displacement of the lens from its anatomical position [**1**]. A partial dislocation refers to the lens as being subluxated, which happens when the zonule is slackened and the lens is only partially within the hyaloid fossa. Complete dislocation or luxation implies the lens being completely torn with its migration, most frequently in the vitreous body [**2**]. Most cases are acquired, usually with trauma in early life, or pseudoexfoliation syndrome, hypermature cataract, or zonulopathies that appear later, therefore usually presenting as unilateral [**3**]. The other underlying cause of the disease includes congenital or development conditions such as Weill-Marchesani syndrome, Marfan syndrome, Homocystinuria within some complex genetic syndromes, or isolated in Ectopia Lentis et Pupillae and Familial Ectopia Lentis [**1**,**2**]. The latter is inherited either in an autosomal dominant pattern, caused by mutations in the FBN1 gene or in an autosomal recessive pattern involving the ADAMTSL4 gene [**4**]. Familial Ectopia Lentis can be present at birth or develop later in life, usually manifesting itself as a bilateral, symmetrical, most often superotemporal dislocation of the lens [**1**]. The main complaint of the patient is loss of visual acuity, which is directly proportional with the dislocation degree, whereas a slit lamp examination can highlight tremulous motion of the iris (iridodonesis) and lens (phacodonesis) when the eye moves, fluctuating between the anterior chamber, pupil block and vitreous that may herniate into the anterior chamber. Possible complications of the disease are cataract, lens-induced uveitis or glaucoma or endothelial touch, which appear earlier than in the general population [**5**]. Together with a significant refractive defect, usually myopia and lenticular astigmatism, these constitute an indication for the surgical removal of the lens. Phacoemulsification in cataract surgery with Ectopia Lentis remains a real challenge for the ophthalmic surgeon, despite the progress related to instrumentation, devices, and surgical techniques [**4**,**5**].

## Case report

**Fig. 1:**
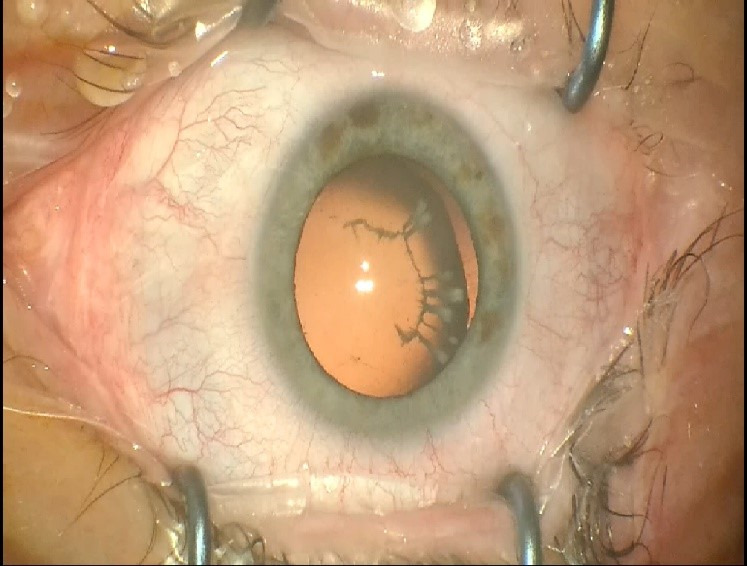
Biomicroscopy revealed cortical and nuclear opacities of the lens in BE and a 180-degree zonular dehiscence with moderate bilateral superotemporal dislocation of the lens

A 48-year-old female patient presented to the clinic with gradual loss of vision during the last year. A BCVA of the RE of 0.16 and LE 0.25; ARM RE: -7,00-0,75/ 5°and LE: -5,25-2,75/ 0° and IOP RE: 17 mmHg, LE: 20 mmHg, were observed. Biomicroscopy revealed cortical and nuclear opacities of the lens in BE and a 180-degree zonular dehiscence with moderate bilateral superotemporal dislocation of the lens (**Fig. 1**). The posterior pole was within normal limits. Specular microscopy of the corneal endothelium of the RE revealed a 2646/ mm3 cell density and LE a 2688/ mm3 cell density.

Ocular biometry was carried out with an Allegro Biograph, showing an axial length at the RE of 24,27 mm and 24,83 for the LE. PC-IOL values were calculated for a -2.5 D refractive target.

No similar cases were reported in the patient’s family history and significant genetic or metabolic conditions were ruled out. Also, the patient denied any ocular trauma. A complete set of blood tests were performed together with a cardiovascular examination that came in within normal limits.

## Surgical technique

**Fig. 2:**
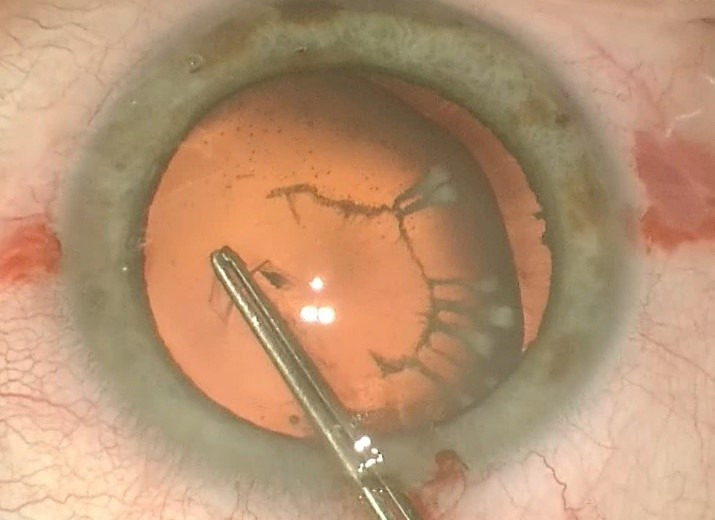
Capsulorhexis was made with a Utrata forceps under viscoelastic substance, Soft Shell Technique

**Fig. 3:**
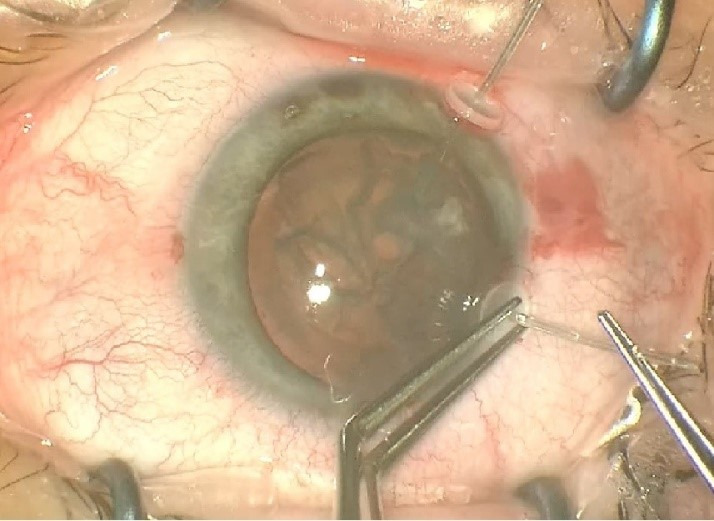
Another 2 side-ports at 7 and 10 o’clock (for the RE) and at 1 and 5 o’clock (for the LE) were made to apply two new capsule retractors to stabilize the bag

**Fig. 4:**
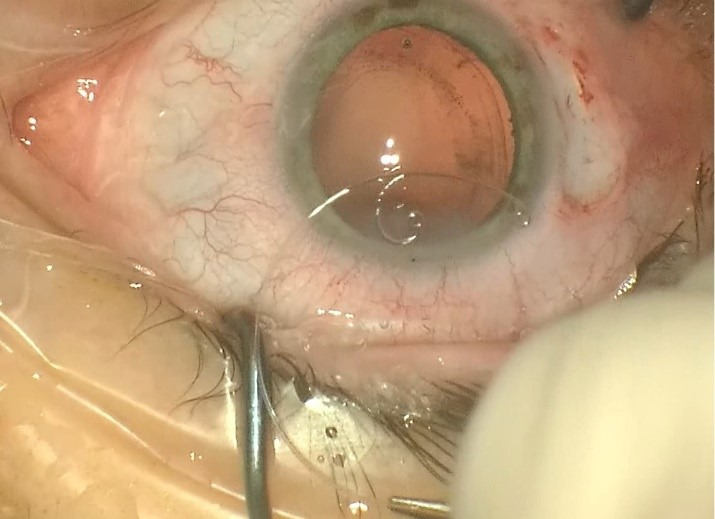
The capsular bag was stabilized using a tension ring, Cionni model

Two similar surgeries were carried out at an interval of 3 weeks between each eye. In preparation for the surgery, the axis of the cylinder was marked at the slit lamp (for the LE) followed by parabulbar anesthesia with lidocaine (2%). The periocular area was disinfected with povidone-iodine and a sterile drape was attached. A 2.2 mm main clear corneal incision at 11 o’clock (120 degrees) and two side ports of 1.2 mm at 3 and 9 o’clock were made. The capsulorhexis was made with a Utrata forceps under viscoelastic substance, Soft Shell Technique (**Fig. 2**). Another 2 side-ports at 7 and 10 o’clock (for the RE) and at 1 and 5 o’clock (for the LE) were made to apply two new capsule retractors to stabilize the bag (**Fig. 3**). After a proper viscodissection, phacoemulsification of the nucleus (using Stop & Chop technique) and cortex (I/A bimanual) was carried out. Subsequently, the capsular bag was stabilized using a tension ring, Cionni model (**Fig. 4**). After the detachment of the conjunctiva towards the limbus, a 3 mm scleral incision was made, through which the prolene 10/0 suture was externalized. The suture was tightened to the point of bag centration and tied; the knot being rotated through the sclerotomy. A monofocal +23.0 PC-IOL was implanted into the capsular bag of the RE whereas a toric +22.0 T3 at 76 degrees axis monofocal was implanted into the LE. The surgery was concluded with the removal of the OVD, stromal hydration of the incisions and the fixation of the conjunctiva to the limbus using diathermocoagulation. The prophylaxis of endophthalmitis was done by subconjunctival injection with Gentamicin and Dexamethasone followed by antibiotic and cortisone ointment and eye patch for 24 hours.

## Results

**Fig. 5:**
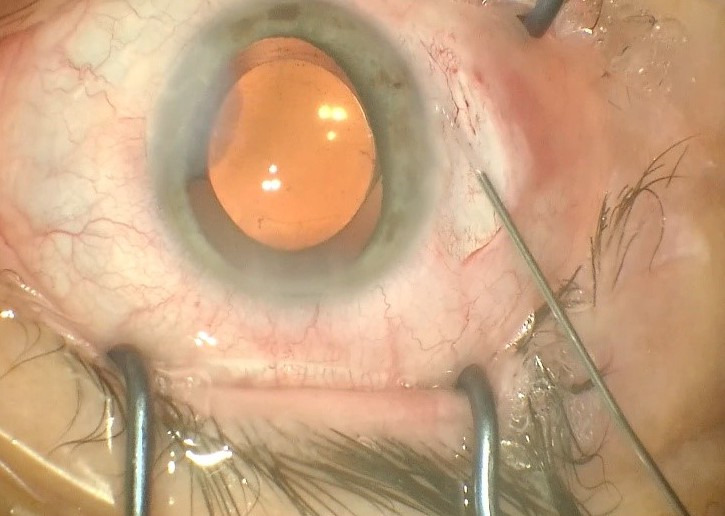
Upon a slit lamp examination of the anterior pole, the cornea was clear, the anterior chamber within normal depth, aphakia with PC-IOL in the capsular bag, well-centered in the optic axis, in the RE

**Fig. 6:**
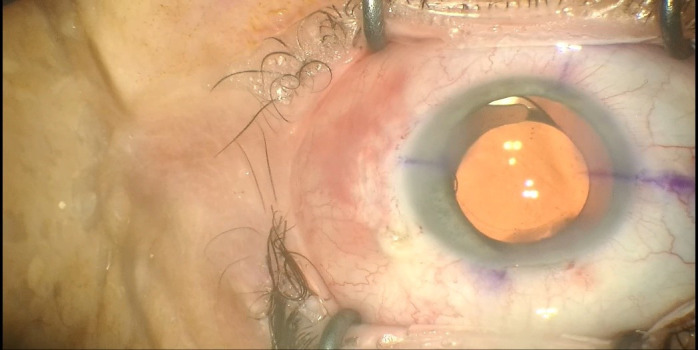
Upon a slit lamp examination of the anterior pole, the cornea was clear, the anterior chamber within normal depth, aphakia with PC-IOL in the capsular bag, well-centered in the optic axis at a 75-degree axis, in the LE

After one week, one month, 3-6-12 months and 2 years postoperatively, the best corrected distance visual acuity was 1, the residual refraction (at the 2 years check-up) showing RE: -2.00/ -1.00x 38 degrees and LE -2.25/ -0.50x146 degrees and IOP measurements were within normal limits. Upon a slit lamp examination of the anterior pole, the cornea was clear, the anterior chamber within normal depth, aphakia with PC-IOL in the capsular bag, well-centered in the optic axis, in the RE (**Fig. 5**) and at a 75-degree axis, in the LE (**Fig. 6**).

## Discussions

Among the things to have in mind when planning cataract surgery in a patient with cataract and Ectopia Lentis are a challenging capsulorhexis, the stabilization of the capsular bag, phacoemulsification of the loose lens and long-time fixation.

Successful capsulorhexis is essential for the preservation of the bag and facile phacoemulsification. An important mention would be the execution direction of the capsulorhexis. It is safer to start pulling away from the area with the intact zonule towards the weakened zonule [**6**]. The capsulorhexis diameter should be around 5 mm to ensure a margin of 2 mm, a generous leaflet for the CTR. 

Using the new capsule retractors, for the subluxated area, ensured safety during phacoemulsification and cortical cleanup. In comparison with the previous models or iris retractors used in cataract surgery with zonular dehiscence, the new retractors maintained the capsular bag in proper position during surgery, no sliding nor tears of the capsulorhexis were experienced. Iris retractors have a short, flexible, and sharp tip that does not properly maintain the equator and can slide off the capsulorhexis margin, making phacoemulsification harder and increasing the risk of capsular tears. Whereas the new retractors, with their length, looped shape and wider angle create a broader contact with the bag and help maintain a distance between the anterior and posterior capsule, which offers better safety.

Phacoemulsification of a loose lens has its challenges as well. Hydrodissection or viscodissection is crucial before starting phacoemulsification, especially in the presence of a zonulopathy, an inadequate hydrodissection may put at risk the remaining zonular fibers. In the case presented, phacoemulsification was performed with lower parameters (aspiration and flow) to reduce turbulence and maintain better control of the fragments. The use of OVD and its periodic augmentation is essential since it maintains a correct anatomy of the bag, which facilitates material evacuation, and decreases the chances of vitreous prolapse [**6**].

CTR can be of great help during surgery, but at the same time it may cause various problems. By applying them, an expended capsular bag is obtained, while providing better support for the zonule and facilitating phacoemulsification. However, some surgeons prefer using it “as late as you can, but as soon as you must” [**7**], since it may block cortical material in the equator, making it difficult to remove it afterwards. The CTR (Cionni model) was used in this case after completing phacoemulsification, this being possible due to the use of the new capsule retractors.

## Conclusion

1. The new capsule retractors maintain the equator of the lens bag, as well as the capsulorhexis margins, compared to the standard iris hook, which does not maintain the equator and which can easily slide off the capsulorhexis margin, making phacoemulsification more difficult and increasing the risk of capsular tears.

2. The capsular tension ring (Ciaonni Model) sutured to the sclera maintained the capsular bag and the PC-IOL well centered two years postoperatively. 

**Conflict of Interest statement**

The authors state no conflict of interest.

**Informed Consent and Human and Animal Rights statement**

Informed consent has been obtained from all individuals included in this study.

**Authorization for the use of human subjects**

Ethical approval: The research related to human use complies with all the relevant national regulations, institutional policies, is in accordance with the tenets of the Helsinki Declaration, and has been approved by the review board of “Constantin Papilian” Military Emergency Hospital Cluj-Napoca, Cluj, Romania.

**Acknowledgements**

None.

**Sources of Funding**

None.

**Disclosures**

None.
